# Risedronate therapy in patients with mild-to-moderate chronic kidney disease with osteoporosis: post-hoc analysis of data from the risedronate phase III clinical trials

**DOI:** 10.1186/s12882-017-0478-9

**Published:** 2017-02-15

**Authors:** Takashi Shigematsu, Ryoichi Muraoka, Toshitsugu Sugimoto, Yoshiki Nishizawa

**Affiliations:** 1 Department of Nephrology, Wakayama Medical University, 811-1 Kimiidera, Wakayama-City, Wakayama 641-8509 Japan; 2 Data Science Group, Clinical Development Department, EA Pharma Co., Ltd, Tokyo, Japan; 3 Internal Medicine 1 Shimane University Faculty of Medicine, Shimane, Japan; 4 Department of Metabolism, Endocrinology and Molecular Medicine, Osaka City University Graduate School of Medicine, Osaka, Japan

**Keywords:** Chronic kidney disease (CKD), Bone disease, Osteoporosis, Biomarkers, Calcium, Treatment

## Abstract

**Background:**

The clinical effect of bisphosphonate treatment has not been clearly evaluated by kidney function in Japanese Chronic Kidney Disease (CKD) patients with osteoporosis. This study analyzed the data from three risedronate Japanese phase III trials. The clinical effect of risedronate therapy was evaluated in CKD patients with osteoporosis.

**Methods:**

The Japanese clinical trials involved 852 subjects who received risedronate (2.5 mg once daily or 17.5 mg once weekly) and whose estimated glomerular filtration rate (eGFR) were calculable and at ≥ 30 mL/min. The subjects were divided into subgroups according to the eGFR level: ≥ 90 mL/min/1.73 m^2^, ≥ 60 to < 90 mL/min/1.73 m^2^, ≥ 30 to < 60 mL/min/1.73 m^2^. Lumbar spine bone mineral density (BMD), bone turnover markers (BTMs) and adverse events were evaluated at 48 weeks.

**Results:**

Adverse event incidence was similar among three subgroups. There was also no exacerbation of impaired kidney function associated with risedronate administration in the subjects with eGFR above 30 mL/min/1.73 m^2^. Risedronate administration induced a significant increase in lumbar spine BMD and significant inhibition of BTMs in three subgroups.

**Conclusions:**

The risedronate therapy showed similar clinical effects in CKD patients with osteoporosis compared to those without CKD.

## Background

There is an overlap in the age of onset of chronic kidney disease (CKD) and osteoporosis. The incidence of these diseases increases proportionally with age. Age is the main factor affecting kidney function. The glomerular filtration rate (GFR), an index of kidney function, decreases linearly with age [1]. The elderly account for a large proportion of osteoporosis patients, many of whom also have CKD. It is therefore important to consider kidney function in patients with osteoporosis. Klawansky et al. examined the relationship between age, kidney function, and bone mineral density (BMD) in patients with osteoporosis and reported impaired kidney function in 85% of the female subjects and 58% of the male subjects [2]. Numerous clinical trials on bisphosphonates have been conducted, although they have excluded patients with impaired kidney function based on serum creatinine levels. Therefore there is only a limited amount of information on the safety and efficacy of bisphosphonate treatment in patients with impaired kidney function or kidney failure [3–5].

Risedronate is a pyridyl compound and a third generation bisphosphonate. It has been demonstrated to be effective for treating osteoporosis [5–11]. In the Japanese population, risedronate has been shown to be an effective treatment as an oral dose of 2.5 mg once daily, 17.5 mg once weekly, and 75 mg once monthly [6–9]. In this study, the dosages used in Japan were half the dosage used outside of Japan (5 mg once daily, 35 mg once weekly) [12]. The plasma concentrations of risedronate after the administration of 2.5 mg risedronate to the Japanese population were nearly comparable to the serum concentrations after the administration of 5 mg risedronate to the UK study population. The difference in oral bisphosphonate dosages between Japanese and subjects outside Japan suggested a difference in bioavailability between Japanese and non-Japanese individuals, although the reasons for this difference remain unknown [13]. As a consequence these doses of risedronate have been approved and can be used clinically. In our study, data from Japanese phase III clinical trials of risedronate were analyzed. The study evaluated the safety and efficacy of risedronate in Japanese osteoporosis patients who were divided into subgroups according to the level of estimated GFR (eGFR): ≥ 90 mL/min/1.73 m^2^, ≥ 60 to < 90 mL/min/1.73 m^2^, ≥ 30 to < 60 mL/min/1.73 m^2^.

## Methods

### Study design

**Fig. 1:**
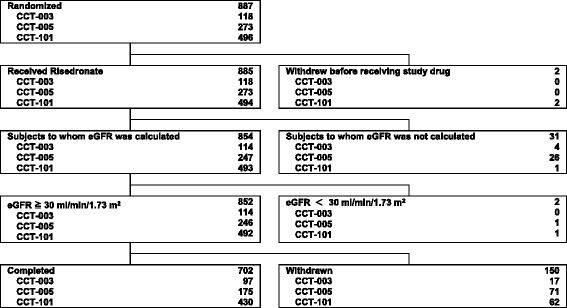
Disposition of subjects

This study analyzed data from three phase III multicenter, randomized, double-blind, controlled trials (CCT-003, CCT-005, and CCT-101). The objective of CCT-003 and CCT-101 trial was to demonstrate the non-inferiority of 2.5 mg daily risedronate to etidronate, the non-inferiority of 17.5 mg weekly risedronate to 2.5 mg daily risedronate on BMD of the lumbar spine, respectively, while the objective of CCT-005 trial was to demonstrate the non-inferiority of 2.5 mg daily risedronate to etidronate on incidence of vertebral fracture. These trials were conducted in Japan from March 1999 to July 2004 [6–8]. The analysis was performed on the data from 887 osteoporosis patients who were assigned to a group receiving risedronate whose dose and administration period varied by trial (Fig. 1).

### Patients

The subjects were of either sex and were patients with involutional osteoporosis based on the diagnostic criteria for primary osteoporosis established by the Japanese Society for Bone and Mineral Research (JSBMR) [14, 15]. Primary osteoporosis was defined by the presence of a fragility fracture and BMD < 80% of the ‘young adult mean’ (20 to 44 years of age), or BMD < 70% of the ‘young adult mean’ in the absence of a detectable fragility fracture [15]. The subjects were aged between 40 and 75 years (inclusive) in the CCT-003 trial, at least 50 years in the CCT-005 and CCT-101 trial. The trial period was 48 weeks in the CCT-003 trial, 96 weeks in the CCT-005 trial, and 48 weeks in the CCT-101 trial. In the CCT-003 and CCT-005 trials, the eligible patients were randomized to receive either treatment with 2.5 mg of risedronate once daily (risedronate 2.5 mg group) or intermittent treatment with etidronate (one cycle: 2 weeks of 200 mg once daily followed by 10 weeks off) (cyclic intermittent etidronate group). In the CCT-101 trial, the eligible patients were assigned randomly to either treatment with 2.5 mg of risedronate once daily (risedronate 2.5 mg group) or treatment with 17.5 mg of risedronate once weekly (risedronate 17.5 mg group). Weekly dosing with 17.5 mg risedronate was well tolerated in Japanese osteoporotic patients, and showed equivalent efficacy to daily dosing with 2.5 mg risedronate [8]. In each trial, all the patients received oral 1.54 g of calcium lactate (200 mg of calcium daily) during the trial period as a supplement for dietary calcium deficiency. The daily dose of calcium was based on the result of the National Nutrition Survey conducted by the Ministry of Health, Labor, and Welfare (recommended daily allowance of calcium for Japanese, 600 mg; actual intake, 585 mg on average in 1995) and on determination of the necessary amount in the elderly, estimated in a calcium balance study (700–800 mg) [16]. The exclusion criteria included patients with any of the following conditions: secondary osteoporosis, disease other than secondary osteoporosis that causes bone loss, findings that affect BMD measurement of the lumbar spine, a history of oral bisphosphonate use within one year of trial commencement, use of oral risedronate, use of any drug affecting bone metabolism (vitamin D_3_ and vitamin K_2_ preparations, and calcitonin analogs, etc.) within eight weeks of trial commencement, severe renal, hepatic, or cardiac impairment, gastrointestinal impairment, predisposition to drug hypersensitivity, current use of an anti-cancer drug for malignant tumor treatment, or a history of radiation therapy in the lumbar spine or pelvic region. During the trial period, the patients were prohibited from concurrent use of any drug affecting bone metabolism.

In our study, the analyses were performed according to kidney function level assessed using eGFR. An equation derived from serum creatinine levels of the Japanese population was used to calculate eGFR. The baseline eGFR value of each patient was calculated, and the patients then divided into eGFR subgroups according to the criteria of the Japanese Society of Nephrology [17, 18]: ≥ 90 mL/min/1.73 m^2^, ≥ 60 to < 90 mL/min/1.73 m^2^, ≥ 30 to < 60 mL/min/1.73 m^2^.

### Endpoints

The following indices were evaluated using comprehensive data from the three trials: percentage change in lumbar spine BMD relative to baseline and the effects on bone resorption markers (urine N-terminal telopeptide of type 1 collagen (NTX) and urine C-terminal telopeptide of type 1 collagen (CTX)) and bone formation marker (serum bone-specific alkaline phosphatase (BAP)). In the CCT-003 and CCT-101 trials, BMD measurements were made for anteroposterior L_2_-L_4_ using a dual-energy X-ray absorptiometry (DXA) system (QDR, XR, or DPX type). The BMD of each patient at each time point were measured using the same DXA system. The measurements were made at trial commencement and 12, 24, 36, and 48 weeks. If the subjects discontinued the trial, the measurements were made at the time of withdrawal.

Safety was evaluated based on the adverse events and change from baseline in kidney function and Calcium/Phosphorus. All adverse events were collected prospectively starting with the first dose of risedronate. Even events that were initially reported by a subject was diagnosed and monitored by investigators. The incidence of overall adverse events, adverse events associated with kidney, urinary, gastrointestinal disorders, osteonecrosis of the jaw and atypical femoral fractures, were analyzed.

### Statistical analyses

The mean ± standard deviation (SD) values of the following variables were calculated for each subgroup: age and body mass index (BMI) at baseline; and eGFR, serum creatinine, calcium, phosphorus, bone turnover markers (urine NTX, urine CTX, and serum BAP) and lumbar spine BMD for each time point. We conducted linear mixed effect model analyses on the efficacy and safety endpoints collected sequentially. The mixed models included subgroup, time, and interaction of subgroup and time as fixed effects, and the subjects as a random effect. The incidence and number of subjects with adverse events were also obtained for each subgroup. We performed the F test for numerical variables and chi-square test for nominal variables in baseline characteristics, and Fisher’s exact test for adverse events. The statistical analyses were performed using SAS version 9.3 (Cary, NC, USA).

## Results

### Patient disposition

A total of 887 eligible patients were randomized to receive risedronate (CCT-003 trial, *n* = 118: CCT-005 trial, *n* = 273; and CCT-101 trial, *n* = 496). The dose and administration period varied in the three trials, although the subjects were not subdivided according to these characteristics in our study. Of the eligible patients, two patients in the CCT-101 trial were excluded because the study drug was not administered. Of the remaining 885 patients, 852 patients had an eGFR that was calculable and at ≥ 30 mL/min, and had their kidney function evaluated (Fig. 1). These 852 patients were the subjects in our study.

The baseline characteristics of the subjects are shown in Table 1 (eGFR ≥ 90 mL/min/1.73 m^2^ group; 99 subjects (11.6%), ≥ 60 to < 90 mL/min/1.73 m^2^ group; 525 subjects (61.6%), ≥ 30 to < 60 mL/min/1.73 m^2^ group; 228 subjects (26.8%), respectively). There were statistically significantly differences among the subgroups in age, daily/weekly, body mass index (BMI).

**Table 1:** Baseline characteristics of subjects

	Overall	eGFR (mL/min/1.73 m^2^)			
		≧90	≧60 to <90	≧30 to <60	*P* value
Number of subjects	852	99	525	228	
Age (years)	68.6 ± 8.1	63.7 ± 7.4	68.0 ± 7.7	72.1 ± 7.7	<0.0001*
Sex (male/female)	32/820	5/94	21/504	6/222	0.5110**
Daily/Weekly	604/248	63/36	350/175	191/37	<0.0001**
BMI (kg/m^2^)	22.2 ± 3.1	21.3 ± 2.8	22.0 ± 2.9	22.9 ± 3.3	<0.0001*
Lumbar spine BMD (g/cm^2^)	0.67 ± 0.09	0.67 ± 0.08	0.67 ± 0.09	0.67 ± 0.08	0.7043*

Values are mean ± SD

*F test, **Chi square test

### Efficacy: BMD and bone turnover markers

**Fig. 2:**
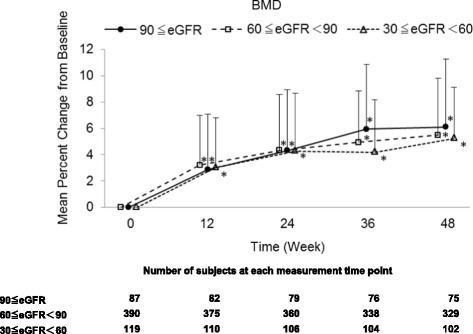
Mean percent change (± SD) from baseline in lumbar spine BMD in receiving risedronate. Patients were stratified into three cohorts by eGFR in baseline, and the mean percent change in lumbar spine BMD in each cohort was shown. *p < 0.001, significantly different from baseline

**Fig. 3:**
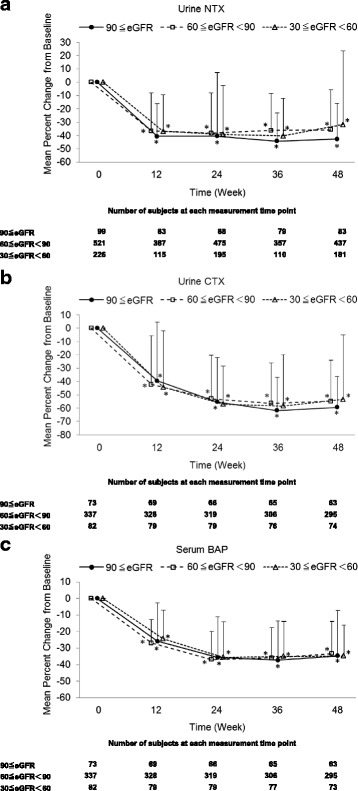
Mean percent change (± SD) from baseline in bone turnover markers (Urine NTX (**a**), Urine CTX (**b**) and Serum BAP (**c**)) in receiving risedronate. Patients were stratified into three cohorts by eGFR in baseline, and the mean percent change in bone turnover markers in each cohort was shown. *p < 0.001, significantly different from baseline

All subgroups showed a significant increase in lumbar spine BMD and a significant inhibition of bone turnover markers (urine NTX, urine CTX, and serum BAP) after 48 weeks of risedronate administration (Figs. 2 and 3). The interactions between time and subgroups were not significant on inhibition of bone turnover markers (NTX; *p* = 0.0759, CTX; *p* = 0.3183, BAP; *p* = 0.7064), but increase in lumbar spine BMD (*p* = 0.0056). But, BMD percent change from baseline at 48 weeks among subgroups were not significant (*p* = 0.5835).

### eGFR, Serum creatinine, calcium, and phosphorus

The interactions between time and subgroups were not significant on calcium (*p* = 0.1129) and phosphorus (*p* = 0.1041), but eGFR (*p* < 0.0001) and creatinine (*p* < 0.0001) (Table 2).

**Table 2:** Mean change from baseline in kidney function and Calcium/Phosphorus and the results of the test of the fixed effects by the linear mixed effect model analyses

		eGFR (mL/min/1.73 m^2^)								
		≧90			≧60 to < 90			≧30 to < 60		
		N	Mean	SD	N	Mean	SD	N	Mean	SD
eGFR (mL/min/1.73 m^2^)	Baseline	99	99.19	9.56	525	72.12	7.98	228	50.74	6.54
	12 weeks	94	91.84	15.90	498	70.77	10.92	209	54.43	10.43
	24 weeks	89	88.89	12.24	482	70.74	11.26	201	54.64	10.41
	36 weeks	86	91.22	13.91	455	71.75	11.65	184	55.54	10.59
	48 weeks	84	93.04	14.08	441	72.11	11.95	177	55.06	11.48
		subgroup					*P* < 0.0001			
		time					*P* < 0.0001			
		interaction of the subgroup and time					*P* < 0.0001			
Creatinine (mg/dL)	Baseline	99	0.48	0.05	525	0.64	0.07	228	0.86	0.12
	12 weeks	94	0.53	0.09	498	0.65	0.10	209	0.82	0.16
	24 weeks	89	0.54	0.08	482	0.65	0.10	201	0.82	0.16
	36 weeks	86	0.53	0.09	455	0.65	0.10	184	0.81	0.15
	48 weeks	84	0.52	0.08	441	0.64	0.11	177	0.82	0.17
		subgroup					*P* < 0.0001			
		time					*P* = 0.0178			
		interaction of the subgroup and time					*P* < 0.0001			
Calcium (mg/dL)	Baseline	99	9.15	0.37	525	9.08	0.41	228	9.05	0.40
	12 weeks	83	9.10	0.39	387	9.10	0.38	115	9.07	0.41
	24 weeks	88	9.13	0.36	481	9.12	0.41	198	9.07	0.40
	36 weeks	79	9.11	0.40	356	9.17	0.41	111	9.21	0.37
	48 weeks	83	9.14	0.35	441	9.13	0.41	182	9.09	0.44
		subgroup					*P* = 0.4597			
		time					*P* < 0.0001			
		interaction of the subgroup and time					*P* = 0.1129			
Phosphorus (mg/dL)	Baseline	26	3.52	0.34	187	3.36	0.47	146	3.43	0.52
	12 weeks	14	3.48	0.43	59	3.29	0.49	36	3.36	0.45
	24 weeks	22	3.28	0.46	162	3.22	0.55	119	3.31	0.48
	36 weeks	14	3.14	0.36	51	3.14	0.48	34	3.31	0.60
	48 weeks	20	3.44	0.45	146	3.32	0.48	109	3.35	0.51
		subgroup					*P* = 0.0888			
		time					*P* < 0.0001			
		interaction of the subgroup and time					*P* = 0.1041			

The linear mixed models included subgroup, time, and interaction of subgroup and time as fixed effects, and the subjects as a random effect

### Safety

There was also no difference between subgroups in the incidence of overall adverse events, adverse events associated with gastrointestinal disorders, or urinary- and kidney function-related adverse events (Table 3). In addition, there were no reported cases of osteonecrosis of the jaw and atypical femoral fracture.

**Table 3:** Summary of adverse events

	eGFR (mL/min/1.73 m^2^)			
	≧90	≧60 to <90	≧30 to <60	*P* value
Number of subjects	99	525	228	
All adverse events	82 (82.8%)	462 (88.0%)	198 (86.8%)	0.3609*
gastrointestinal symptoms–relate adverse events	33 (33.3%)	192 (36.6%)	88 (38.6%)	0.6631*
Abdominal pain upper	4 (4.0%)	31 (5.9%)	7 (3.1%)	NT
Constipation	4 (4.0%)	37 (7.0%)	19 (8.3%)	NT
Diarrhoea	4 (4.0%)	17 (3.2%)	12 (5.3%)	NT
Stomach discomfort	11 (11.1%)	44 (8.4%)	14 (6.1%)	NT
Urinary- and kidney function-related adverse events	2 (2.0%)	11 (2.1%)	7 (3.1%)	0.6295*
Osteonecrosis of the jaw	0 (0.0%)	0 (0.0%)	0 (0.0%)	NT
Atypical femoral fractures	0 (0.0%)	0 (0.0%)	0 (0.0%)	NT

*NT* not test

* Fisher’s exact test

## Discussion

In the present study, we have found that treatment with risedronate in Japanese osteoporosis patients irrespective of the level of eGFR was effective.

In this study, there were some differences among the subgroups (Table 1). But these differences were considered clinically insignificant. Because age was included in the calculation formula for eGFR, differences in age among the subgroups was not unusual. This relationship was also observed in other studies [19, 20]. The efficacy and safety were similar between daily dosing with 2.5 mg risedronate and weekly dosing with 17.5 mg risedronate [8]. Therefore the difference did not affect the current evaluation. BMI values were within the normal range (18.5-25.0 kg/m^2^), and did not indicate obesity.

Based on other studies, risedronate is a safe and effective drug for the treatment and prevention of osteoporosis [5–11]. But, similar to other bisphosphonates, risedronate is excreted through the kidneys. It may therefore accumulate in the kidneys of patients with kidney insufficiency [19]. Therefore, the effect of renal insufficiency on the safety and efficacy of risedronate treatment is an important consideration in clinical practice. There was a report about the safety and efficacy of risedronate in the osteoporosis patient including eGFR < 30 ml/min [19], but there is no report about Japanese patients. In Japan, the package insert of risedronate states that it is contraindicated in patients with severe kidney insufficiency (creatinine clearance of less than approximately 30 mL/min). Other bisphosphonates are also carefully administered, and bisphosphonates should therefore be administered carefully in patients with kidney insufficiency. In our study, changes from baseline in eGFR and serum creatinine after 48 weeks of treatment were observed. The chronological changes in eGFR and creatinine measurements observed in this study are thought to be due to regression to the mean (phenomenon in which variables used at varied distributions at baseline or sub-group means for variables that are strongly correlated to such variables will approach the overall mean at next measurement) rather than risedronate administration, and when we took into consideration the fact that the changes from baseline were small, we concluded that these changes were clinically insignificant. The changes in serum calcium and phosphorus were small and there were no significant differences. Miller et al. found that although there were sporadic statistically significant differences between the treatment groups with respect to the mean percent changes from baseline in serum calcium and serum phosphorus, these differences were small and an expected consequence of changes in calcium homeostasis caused by inhibition of bone resorption during therapy. They were therefore not considered to be clinically meaningful [19]. Yanik et al. conducted a trial on women with osteoporosis or osteopenia and reported that blood urea nitrogen, serum creatinine, and eGFR did not change from baseline following 12 months of risedronate treatment [21]. These results are also consistent with ours.

We found that there was no difference in the incidence of overall adverse events, adverse events associated with gastrointestinal disorders, or urinary- and kidney function-related adverse events among the subgroups regardless of the level of eGFR. Our findings are consistent with subsequently published analyses [19, 20]. Miller et al. reported that there was no difference in the incidence of overall adverse events or renal-related events of risedronate treatment by eGFR, Jamal et al. also reported that there was no difference in the frequency of gastrointestinal events in women with reduced renal function (4.5%) compared with those without (5.2%; *p* = 0.5), and renal adverse events in women with reduced renal function (2.1%) compared with those without (2.3%; *p* = 0.68).

Kaji et al. found a higher rate of vertebral fracture in postmenopausal women at CKD stage 3 or greater compared with those at stage 1 or 2. They also examined the relationship between creatinine clearance and vertebral fracture in CKD stage 2 postmenopausal women using logistic regression analysis with adjustment for years since menopause and lumbar spine BMD. The creatinine clearance level was shown to be associated with the risk of vertebral fracture in these women [22]. Therefore, treatment for osteoporosis is important in patients who also have CKD [4]. In Japanese osteoporosis patients with decreased kidney function, evaluation by kidney function has not always been performed when investigating the safety and efficacy of drugs used to treat osteoporosis, despite the clinical importance of these characteristics. Our data are important because Japanese osteoporosis patients have been shown to have some degree of kidney impairment, and there is currently little published information about the effect of risedronate treatment in patients with kidney insufficiency. The results of our study showed that lumbar spine BMD increased and bone turnover markers (urine NTX, urine CTX, and serum BAP) were inhibited regardless of kidney function after 48 weeks of risedronate administration. There was no significant difference in the magnitude of the increase in lumbar spine BMD or decrease in bone turnover markers among these subgroups. In a study conducted outside of Japan, Miller et al. evaluated comprehensive data from nine risedronate phase III studies and examined the safety and effect of the drug on kidney function in osteoporosis patients (severe kidney impairment: creatinine clearance (CrCl) < 30 mL/min, moderate kidney impairment: CrCl > 30 to < 50 mL/min, and mild kidney impairment: CrCl > 50 to < 80 mL/min). Lumbar spine BMD increased significantly and the incidence of new vertebral fractures decreased significantly in the group receiving risedronate (regardless of kidney function) compared with the group receiving placebo [19]. They also reported that comparison of the placebo group and subgroups receiving risedronate showed all subgroups had a greater reduction in bone turnover marker levels (serum BAP and urine deoxypyridinoline levels) relative to baseline levels. These results of Miller et al. were consistent with our results.

There were some limitations in this study. First, in this study did not include data on bisphosphonate administration in osteoporosis patients with kidney function of eGFR < 30 mL/min/1.73 m^2^. Further studies are therefore necessary. Second, this was a post hoc analysis of pooled data from 3 trials, which were originally neither intended to determine the influence of kidney function nor randomized for the level of eGFR. Third, this study did not analyze the samples with the intention to treat, and excluded some patients. Fourth, this study did not assess the effect of risedronate on hip BMD, and did not evaluate the anti-hip fracture efficacy of risedronate due to lack of data. Finally, this study only investigated a 48 weeks period. Adverse events such as osteonecrosis of the jaw and atypical fractures are likely to require long-term observation, which means that this study was not able to sufficiently examine for these adverse events.

## Conclusions

In our study, the data from Japanese phase III trials conducted on osteoporosis patients was analyzed. The results showed that risedronate therapy has similar clinical effects in CKD patients with osteoporosis compared to those without CKD.

## Abbreviations

CKD: Chronic Kidney Disease
eGFR: Estimated glomerular filtration rate
BMD: Bone mineral density
BTMs: Bone turnover markers
GFR: Glomerular filtration rate
JSBMR: Japanese Society for Bone and Mineral Research
NTX: N-terminal telopeptide of type 1 collagen
CTX: C-terminal telopeptide of type 1 collagen
BAP: Bone-specific alkaline phosphatase
DXA: Dual-energy X-ray absorptiometry
SD: Standard deviation
BMI: Body mass index
CrCl: Creatinine clearance.
